# Assessment of quality of life, information, and supportive care needs in patients with muscle and non-muscle invasive bladder cancer across the illness trajectory

**DOI:** 10.1007/s00520-019-4649-z

**Published:** 2019-02-14

**Authors:** Jiil Chung, Girish S. Kulkarni, Robin Morash, Andrew Matthew, Janet Papadakos, Rodney H. Breau, David Guttman, Jackie Bender, Jennifer M. Jones

**Affiliations:** 1 Cancer Rehabilitation and Survivorship Program, Princess Margaret Cancer Centre, Toronto, Canada; 2 Department of Surgery, Division of Urology, University Health Network and University of Toronto, Toronto, Canada; 3 Wellness Beyond Cancer Program, The Ottawa Hospital, Ottawa, Canada; 4 Departments of Surgery and Supportive Care, Princess Margaret Cancer Centre, Toronto, Canada; 5 Cancer Education Program, Princess Margaret Cancer Centre, Toronto, Canada; 6 Division of Urology, The Ottawa Hospital and University of Ottawa, Ottawa, Canada; 7 Bladder Cancer Canada, Toronto, Canada

**Keywords:** Bladder cancer, Supportive care needs, Patient education, Cancer survivorship, Informational needs, Quality of life

## Abstract

**Background:**

To date, little research has examined the quality of life and cancer-associated needs of bladder cancer patients. The objective of the current study was to assess the quality of life (QoL), informational needs, and supportive care needs (SCN) in a large sample of muscle invasive (MIBC) and non-muscle invasive (NMIBC) bladder cancer survivors across the treatment trajectory (newly diagnosed and undergoing treatment, post-treatment follow-up, and treatment for advanced/recurrent disease).

**Methods:**

Questionnaires were distributed to a convenience sample of patients registered with Bladder Cancer Canada, the Princess Margaret Cancer Centre, or The Ottawa Hospital. Eligibility criteria included being > 18 years of age, English-speaking, and diagnosed with bladder cancer. The questionnaire included an adapted tool to measure informational needs, and validated measures for QoL (Bladder Utility Symptom Scale, BUSS) and SCN (Cancer Survivors’ Unmet Needs Measure, CaSUN). QoL scores and unmet needs were calculated and compared between disease groups and cancer trajectory groups.

**Results and limitations:**

Of the 1126 surveys distributed, 586 were completed (response = 52%). Mean age was 67.3 ± 10.2 years, and 401 participants (68.7%) were male. The mean QoL score (BUSS) for the sample was 78.1 ± 17.9 (median 81.7). Respondents with MIBC had significantly lower QoL scores compared to NMIBC. Further, scores differed across the cancer phase groups with the follow-up surveillance group having significantly higher QoL scores compared to the newly diagnosed and advance/recurrent disease groups. The ten most highly ranked informational needs were from the medical, physical, and practical domains. Eighty-eight percent (95% CI 85–91%) of respondents reported at least one SCN, with a median of 12. Over half of the participants (54%, 95% CI 49–59%) had at least one unmet need and 15% had ≥ 10 unmet needs. Newly diagnosed participants had the highest number of unmet needs.

**Conclusion:**

We found that the number of unmet supportive care needs and quality of life differed across cancer trajectory and disease groups. Future efforts should focus on the development and evaluation of tailored resources and programs to address the needs of people diagnosed and treated for BC.

**Electronic supplementary material:**

The online version of this article (10.1007/s00520-019-4649-z) contains supplementary material, which is available to authorized users.

## Background

Bladder cancer (BC) is the second most common genitourinary cancer in North America and accounts for an estimated 4.5% of all new cancer cases worldwide [1]. The largest risk factors for BC are smoking, being male, and age, and the prevalence is expected to significantly increase over the next two decades due to the aging population [2].

The diagnosis and treatment of BC are stressful events associated with numerous supportive care needs (SCN) that continue beyond treatment [3–11]. However, a 2009 survey of BC specialists (*n* = 43) in North America found that there were limited resources directed to BC survivors including support groups, survivorship programming, community services, and patient navigation/information, and that providers lacked awareness of available resources [12]. Further, there is very little research on the informational needs and SCN of both NMIBC and MIBC survivors; which may in part explain the lack of appropriate and tailored resources directed to them [10, 11]. In addition, the quality of life (QoL) of BC patients has not been well-described despite the complexities of treatment and its chronic nature, and while generic BC-specific measures of QoL have been developed, such as the EORTC QLQ’s, BCI, and FACT-Bl, these instruments have limitations including being restricted to subsets of BC patients, missing important domains for patients, and lacking evidence on psychometric properties [13–16]. Specific patient-driven global BC QoL scales that include attributes specific to MIBC and NMIBC have only recently been developed [17].

There remains a need for larger, more comprehensive studies that can inform appropriate person-centered care activities for BC patients, including the development and tailoring of appropriate patient education and supportive care resources and services. Consequently, we conducted a large cross-sectional descriptive study to describe the QoL and informational and supportive care needs of NMIBC and MIBC survivors across the cancer care trajectory.

## Materials and methods

### Participants

BC patients were recruited from the Genitourinary Clinic at Princess Margaret (PM) Cancer Centre, the Urology Clinic at The Ottawa Hospital (TOH), and through Bladder Cancer Canada, a national non-profit organization that seeks to support BC survivors and increase awareness of BC among the general public and medical community. Patients were eligible if they were > 18 years of age, could understand and read English, and had been diagnosed with any stage of NMIBC/MIBC. The study was approved by Research Ethics Boards at the University Health Network and The Ottawa Hospital. Participants were informed that completion of the questionnaire implied consent.

### Procedures

Princess Margaret Cancer Centre and The Ottawa Hospital: Patients diagnosed with BC attending clinical appointments were invited to the study and provided the questionnaire package to complete in clinic or return in a pre-paid envelope. Bladder Cancer Canada (BCC): Registered patient members were emailed information regarding the study with a link to the questionnaire that was hosted on FluidSurveys. The four-point survey approach based on Dillman’s Tailored Design Method [18, 19] was used to reduce non-response, which involved sending all registered members an initial e-mail and non-responders up to three follow-up emails. Participants could complete the survey online or by mail.

### Study measures

Participants completed a one-time questionnaire that was developed with input from the research team. The questionnaire package was field tested with five randomly selected BC patients attending routine visits to PM. The field test results were reviewed to assess the time it took participants to complete the questionnaire and to identify any issues with completion of the questions. Completion of the survey implied consent. The questionnaire included the following: (1) Demographic and disease and treatment information, which contained questions about age, sex, residence setting, employment status, and comfort with using the internet. Clinical information included type(s) of treatment received, time since diagnosis, stage and type of bladder cancer at diagnosis, and the phase of cancer treatment they identified with, which were categorized into newly diagnosed, follow-up surveillance, and receiving treatment for recurrent or metastatic disease; (2) Quality of life assessed using the Bladder Utility Symptom Scale (BUSS) [10] which is a global health related QoL questionnaire that evaluates generic and BC-specific domains of QoL, including physical changes such as fatigue, bowel, and bladder function. The BUSS has been validated and was developed in consultation with BC patients and experts and included conceptual framework development, item generation and reduction, question design, and pilot testing. The BUSS is self-administered and includes ten questions and a score out of 100 is given to each participant, with higher scores indicating better QoL. In addition, overall health is measured using a 0–100 visual analogue scale [16]; (3) Informational needs assessed using an internally designed, non-validated questionnaire that was developed based on existing literature regarding patient information and supportive care needs [20]. This tool has been used in other cancer populations and can be easily adapted to the population being studied [21–23]. For the current study, we included 30 relevant items based on consensus from experts (and removed 12 items deemed not applicable). The measure assesses informational needs in the following domains: medical (5 items), practical (6 items), physical (8 items), social (3 items), emotional (7 items), and spiritual (1 item) [13]. Items are measured on a 5-point Likert scale, and participants rate the importance of each item. Scores for each domain were transformed to a 0–100 scale; and (4) Supportive care needs assessed using the Cancer Survivorship Unmet Needs (CaSUN) tool [24], a 35-item validated tool that assesses met and unmet SCN over the preceding month. For each item, respondents indicate whether the particular need is “not needed/not applicable,” “met,” or “unmet.” The items map to five domains: information (IN) (3 items), comprehensive cancer care (CCC) (6 items), existential survivorship (ES) (14 items), quality of life (QOL) (2 items), and relationships (REL) (3 items). There are also six additional positive change (PC) items, and one open-ended question [24]. The number of total needs (met and unmet) and unmet need are summed for all items (range 0–35) and for each domains.

### Data analysis

Descriptive statistics were calculated to provide summary information about the participants and for each outcome measure, and examined for non-normality (kurtosis and skewness). BUSS total scores were not normally distributed and were compared between disease groups (MIBC vs. NMIBC) using Mann-Whiney *U* test, and between cancer trajectory groups (newly diagnosed and undergoing treatment vs. follow-up surveillance vs. treatment for recurrence or metastatic disease) with Kruskal-Wallis test. The proportion of items rated “important” or “very important” in each informational need domain was alculated with 95% confidence interval (CI), and compared across disease groups and cancer phase groups using chi-squared analyses. Proportion (+ 95% CI) of total and domain-specific SCN (No. answering “Need was fully met” or “Need was not fully met”/total no. responses for that item) were calculated and examined. Proportion of “Unmet needs” was calculated for each item (No. answering “Need was not fully met”/No. who identified need (either met/unmet)). SCN total and domain-specific met or unmet (±SD) needs were non-normally distributed and compared across disease groups and cancer phase groups using Mann-Whitney *U* and Kruskal-Wallis tests. Data analyses were performed using the Statistical Package for the Social Sciences (SPSS) version 21.0 with *p* values of < 0.05 considered statistically significant. Bonferroni correction was made for multiple comparisons.

## Results

A total of 586 surveys were completed between November 2014 and April 2016 including *n* = 204 (204/308, 66%) from PM, *n* = 129 (129/183, 70%) from TOH, and *n* = 253 (253/625, 40%) from BCC. The QoL (*p* = 0.428), informational needs (across domains *p* = 0.1 to *p* = 0.95), and SCN scores (met and unmet needs) (*p* = 0.09) were not statistically different across the three samples (Princess Margaret, Ottawa Hospital and BCC). Consequently, the data was pooled together for analyses.

### Demographics and clinical characteristics

The majority of participants were male (68%), born in Canada (68%), had at least post-secondary education (65%), and were very comfortable receiving health information in English (94%). Fifty-seven percent of participants were initially diagnosed with NMIBC, 23% with MIBC, and 18% did not know their diagnosed disease type. At the time of the survey, 13% were newly diagnosed and receiving primary therapy, 66% had completed treatment and were in post-treatment follow-up, and 15% were receiving treatment for recurrent or metastatic disease. Demographic and clinical data are summarized in Table 1.

**Table 1:** Demographic information and clinical characteristics

Variables	Study population (*n*, %) *N* = 586
Age, mean ± SD	67.3 ± 10.2
Sex	
Male	401, 68.4%
Female	183, 31.2%
Country of birth	
Canada	397, 67.7%
Outside of Canada	183, 31.2%
Education	
≤ Post-secondary	204, 34.8%
> Post-secondary	378, 64.5%
Residence setting	
Urban	319, 54.4%
Suburban	166, 28.3%
Rural	97, 16.6%
Time of diagnosis	
0–2 years ago	75, 12.8%
2–5 years ago	288, 49.1%
5 + years ago	190, 32.4%
Disease type	
MIBC	134, 22.9%
NMIBC	337, 57.5%
I do not know	103, 17.6%
TNM staging	
Ta, CIS, T1	324, 55.3%
T2–T4	129, 22.0%
N+, M+	8, 1.4%
I do not know	113, 19.3%
Treatment	
Surgery only	170, 29.7%
Chemotherapy only	16, 2.8%
Immunotherapy (BCG) only	33, 5.8%
Radiation only	3, 0.5%
Surgery + chemotherapy	108, 21.2%
Surgery + immunotherapy (BCG)	261, 51.2%
Surgery + radiation	35, 6.9%
Cancer journey status	
Newly diagnosed or receiving treatment for newly diagnosed cancer	77, 13.1%
Post-treatment follow-up surveillance	388, 66.2%
Diagnosed with and treated for recurrent or metastatic disease	87, 14.8%

*N*, number; *SD*, standard deviation; *MIBC*, muscle invasive bladder cancer; *NMIBC*, non-muscle invasive bladder cancer; *CIS*, carcinoma in situ; *BCG*, Bacillus Calmette-Guérin

### Quality of life

The mean QoL score (BUSS) for the sample was 78.1 ± 17.9 (median 81.7). Respondents with MIBC had significantly lower scores (median 75) compared to NMIBC (median 85) (*U* = 9497, *p* < 0.001). Further, scores differed across the cancer phase groups with the follow-up surveillance group having significantly higher QoL scores (median 83.3) compared to the newly diagnosed (median 72.5) and the recurrent/metastatic groups (median 74.6) (H(2) = 20.71 *p* < 0.001).

### Informational needs

The percentage of items rated “important” or “very important” varied across the domains: medical (95%, 95% CI 94–97%), physical (86%, 95% CI 84–88%), practical (77%, 95% CI 75–80%), emotional (67%, 95% CI 63–70%), social (61%, 95% CI 58–64%), and spiritual (40%, 95% CI 35–44%). The ten most highly ranked informational needs were from the medical [5], physical [3], and practical [2] domains (Table 2).

**Table 2:** The ten most important informational needs (order of the items was determined using all participants)

Informational needs	Proportion of participants with MIBC who found the item important or very important (%)^a^	Proportion of participants with NMIBC who found the item important or very important (%)^a^	Proportion of all participants who found the item important or very important (%)^a^	Domain
1. General information about cancer	89	91	88	Medical
2. Possible side effects of treatment	86	93	88	Medical
3. How to manage side effects of treatment	89	91	88	Medical
4. Treatments advantages/disadvantage	87	89	86	Medical
5. Which symptoms to monitor and report in the future	85	88	86	Physical
6. Further medical tests after treatment	87	85	84	Medical
7. How often to visit the doctor	82	84	82	Practical
8. Drug coverage options	78	76	75	Practical
9. Expected pace of recovery	76	72	72	Physical
10. How to manage changes to memory and attention	72	69	70	Physical

^a^Proportions were calculated by combining the “Very Important” and “important” responses, and dividing by the total number of responses for each item

The MIBC group and the NMIBC group had similar informational needs, with the exception of the practical domain needs, which were ranked higher in the NMIBC group (*p* = 0.046). Across the cancer phase groups, the informational needs were also similar, with the exception of the practical domain items. They were ranked less important by respondents with recurrent or metastatic disease compared to those with newly diagnosed disease (*p* = 0.031) or receiving follow-up surveillance (*p* = 0.035).

### Supportive care needs

Eighty-eight percent (95% CI 85–91%) of respondents reported at least one SCN with a median of 12 and a mean of 15 ± 12.2 (out of a total of 35). The most common SCN were in the comprehensive cancer care (CCC) domain with 82% (95% CI 78.8–85.2%) of respondents reporting a need, followed by 65% (95% CI 61.0–68.9%) in the existential survivorship (ES) domain, 55% (95% CI 50.9–59.1%) in the information (IN) domain, 47% (95% CI 42.3–51.2%) in the quality of life (QOL) domain, and 35% (95% CI 31.1–38.9%) in the relationships (REL) domain. The top 10 SCN (met or unmet) are listed in Table 3 (see Supp Table 1 for all SCN).

**Table 3:** The ten most commonly endorsed supportive care needs (met or unmet) in the CaSUN (order of the items was determined using all participants)

Item	Endorsing^a^ MIBC (%)	Endorsing^a^ NMIBC (%)	Endorsing^a^ total (%)	Domain
1. The very best medical care	70	68	68	CCC
2. To feel like I am managing my health together with the medical team	71	68	68	CCC
3. Concerns regarding my care to be properly addressed	71	68	67	CCC
4. To know that all my doctors talk to each other to coordinate my care	69	61	64	CCC
5. Information provided in a way that I can understand	54	53	52	IN
6. Local healthcare services	54	50	50	CCC
7. Help to manage my concerns about the cancer coming back	50	46	47	ES
8. Up-to-date information	50	46	46	IN
9. Help to manage ongoing symptoms and side effects	50	42	44	QOL
10. Emotional support	53	35	39	ES

*CaSUN*, Cancer Survivors’ Unmet Needs measure; *CCC*, comprehensive cancer care; *IN*, information; *ES*, existentialsurvivorship; *QOL*, quality of life

^a^Percent endorsing the item was calculated by combining the number of responses that identified each item as met or unmet, divided by the total responses for the item

**Fig. 1:**
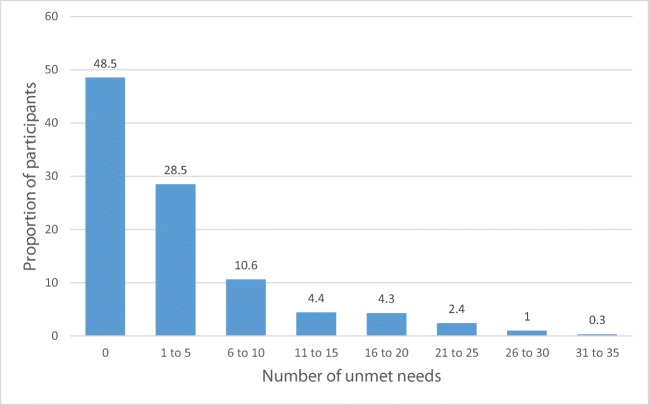
Proportion of participants reporting unmet needs. Proportion of study participants by number of unmet supportive care needs. The number of unmet needs were grouped into ranges of five. Labels represent the proportion of the entire study population that had the corresponding ranges of unmet supportive care needs

The median number of unmet needs was 1.0 with a mean of 4.2 ± 6.7. Over half of the participants (54%, 95% CI 49–59%) had at least one unmet need (Fig. 1). The ES domain had the highest proportion of participants who had at least one unmet need (38%, 95% CI 33.5–42.5%), followed by the CCC domain (34%, 95% CI 29.7–37.0%), REL domain (23%, 95% CI 19.1–26.9), QOL domain (18%, 95% CI 14.5–21.5%), and the IN domain (13%, 95% CI 9.9–16.1%). The ten most common unmet SCN are reported in Table 4 (see Supplementary Table 1 for all unmet SCN).

**Table 4:** The ten most commonly unmet supportive care needs in the CaSUN (order of the items was determined using all participants)

Item	Endorsing as unmet^a^ MIBC (%)	Endorsing as unmet^a^ NMIBC (%)	Endorsing as unmet^a^ total (%)	Domain
1. Help to address problems with my/our sex life	60%	81%	76%	Rel
2. Help to try to make decisions about my life in the context of uncertainty	63%	74%	71%	ES
3. Help to cope with others not acknowledging the impact that cancer has had on my life	67%	72%	69%	ES
4. Help to cope with expectations of me as a cancer survivor	53%	68%	67%	ES
5. Help to cope with changes to my belief that nothing bad will ever happen in my life	61%	67%	64%	ES
6. Help developing new relationships after the cancer	67%	57%	62%	ES
7. Help to find out about financial support or governmental benefits to which I am entitled	53%	66%	60%	PC
8. More accessible hospital parking	58%	58%	60%	CCC
9. Help to deal with the impact that cancer has had on my relationship with my partner	62%	59%	59%	Rel
10. Help getting life and/or travel insurance	70%	57%	59%	PC

*CaSUN*, Cancer Survivors’ Unmet Needs measure; *Rel*, relationships; *ES*, existential survivorship; *PC*, positive changes; *CCC*, comprehensive cancer care

^a^Proportion represents total number who indicated need was not fully met divided by total number who endorsed the need

The MIBC group had median of 1.0 unmet need (mean 4.6 ± 6.5), and the NMIBC group also had a median of 1.0 unmet need (mean of 4.2 ± 6.8) (*U* = 13,549.00, *p* = 0.28). The number of unmet needs differed across the cancer journey groups (H(2) = 17.68, *p* < 0.001). Newly diagnosed participants had a median of 3.5 unmet needs; participants in post-treatment follow-up had a median of 0.0 unmet needs, and those in the metastatic or recurrent cancer had a median 1.5 unmet needs.

Fourteen percent (82/586) of respondents had ≥ 10 unmet needs with a median of 16.0 unmet SCN (mean 17.4 ± 5.8). This group of patients were more likely to be newly diagnosed with (25% vs. 13%, *p* = 0.018) and had lower total BUSS score (60.3 ± 21.1) compared to respondents who had < 10 unmet SCN (79.6 ± 14.7, *p* < 0.001).

## Discussion

This study provides a comprehensive cross-sectional description of the QoL, informational needs, and SCN of BC survivors. It also includes large samples of both MIBC and NMIBC patients at all phases of the cancer trajectory.

QoL was found to be lower in patients with MIBC compared to NMIBC, and in patients who were newly diagnosed or had metastatic or recurrent disease when compared to those who finished treatment and were in long-term follow-up. These findings are not surprising given that more advanced disease requires more complex treatments that may result in a decline in functional health and increased symptom burden along with psychosocial and existential issues related to death and dying [25, 26], and highlight the need for early intervention with palliative programs and services. In addition, the diagnostic and primary treatment phases can be difficult due to acute treatment-related effects [27]. In a recent systematic review of the qualitative evidence on the experience of being diagnosed and treated for bladder cancer, Edmonston et al. describe patient initial shock and fear at diagnosis, subsequent challenges when making treatment decisions including the patient’s struggle to understand treatment options and potential side effects, and the need for support in managing acute side effects of treatment and development of self-management skills [3]. It was encouraging that QoL scores are significantly higher in those who were receiving posttreatment follow-up care, a finding that may suggest that QoL impairments may improve once treatment ends. This finding is supported by qualitative data which describes QoL in BC survivors as initially worse for some patients as they struggle to adapt to a new normal [28] but slowly improves over time as patients begin to feel better and adapt to a “new normal” despite long-term effects of their cancer [29, 30].

In terms of informational needs, we found that the highest-rated domain was the medical domain, which included general knowledge about their cancers, treatment options and side effects, and subsequent post-treatment tests. This result is consistent with findings reported in other cancer patient populations [22, 23]. In a recent qualitative study of MIBC patients, Mohamed et al. found that informational needs of newly diagnosed participants were focused on treatment options and the management of side effects. For postoperative participants, the most important informational needs related to returning to physical and social activities and dealing with worry and fears of cancer recurrence [11]. We found that respondents with MIBC and those who were earlier on in the cancer trajectory placed higher importance on practical information, which includes drug coverage options, home services, and the frequency of hospital visits, maybe due to differences in disease severity and complexity of treatments associated with MIBC, as well as the lack of familiarity with the healthcare system and available resources when one is newly diagnosed.

SCN were also common, with 86% of respondents indicating at least one supportive care need and a median of 12. Most SCN centered on the provision of comprehensive cancer care, such as “feeling like I am managing my health together with the medical team”, and “their care is properly addressed”. Encouragingly, the majority of these needs were met. Approximately half (54%) of the respondents reported ≥ 1 unmet need, and the mean number of unmet needs was lower than the average reported in other cancers [31, 32]. The ES domain, which is primarily psychosocial in nature, addresses needs such as, “concerns of recurrence,” “help with changes in optimism,” and “moving on in life.” It had the highest proportion of unmet needs, with 38% of respondents indicating at least one unmet need in this domain. This finding has been reported in other cancer populations [33, 34]. Patients who were newly diagnosed had higher levels of unmet needs, despite being the group that would see healthcare providers most frequently. The group of respondents with ≥ 10 unmet needs also had a higher proportion of newly diagnosed patients than those who had < 10 unmet needs (25% vs. 13%) and significantly lower quality of life scores. These observations reinforce the need to improve the quality of care for patients early on in their treatment phase, including supporting patients through the decision making process and preparation for treatment and providing open and supportive communication [11, 28, 35, 36].

The findings of this study need to be interpreted in the context of its limitations. This was a cross-sectional study, which does not allow for the examination of cause-effect relationships. In addition, this study was restricted to English-speaking residents in Canada and may not be representative of non-English speaking individuals, or those outside of Canada. We did not address some demographic questions regarding income or caregiver status, which may have had impacts on needs and QoL of the participants. In addition, approximately 2/3 of the study population had a post-secondary education which may be due to the online nature and questionnaire-based nature of the study. This may impact the representation of these findings to the entire BC patient population and potentially result in an underestimation of need. Furthermore, the study cohort may have a larger proportion of younger patients due to the online approach for the BCC population, which can impact the representation of these findings to the entire BC patient population. The BCC group was younger than the PM and OCC cohorts. The use of both online and offline questionnaires may also partially explain the lower response rates seen in the online cohort, as there was no personal interaction and waiting time in the clinic with BCC respondents as there were in the TOH and UHN sites. However, since the informational needs, SCN, and QoL scores did not differ significantly across the three sites, the different response rates likely did not affect our analyses. Despite these limitations, this study provides valuable insight into the QoL, informational needs, and SCN of BC patients. It also emphasizes the need for the development of targeted educational resources, formalized education pathways, and screening for and addressing SCN, as they are common and significantly impact QoL.

## Conclusion

This work provides the initial groundwork to improve person-centered care provided to BC patients. Healthcare professionals play an important role in the provision of patient education and psychosocial support at all phases of treatment, as they are often the patients’ first point of contact. While there are clear benefits in providing appropriate and effective patient education and psychosocial care [37, 38], patients may be reluctant to disclose their SCN with their oncologists. These barriers and gaps impede patients from receiving the required comprehensive care that addresses their needs [39]. These findings highlight the need to develop a standardized approach to the assessment and provision of supportive care that is embedded within care pathways [40]. Future efforts should focus on the development and evaluation of tailored resources and programs to address the needs of people diagnosed and treated for BC.

## Electronic supplementary material

ESM 1 (DOCX 21 kb)
