# Supplementary material for: Assessment of quality of life, information, and supportive care needs in patients with muscle and non-muscle invasive bladder cancer across the illness trajectory
Source: Support Care Cancer. 2019 Feb 14;27(10):3877–85. doi: 10.1007/s00520-019-4649-z (PMC6726665; doi:10.1007/s00520-019-4649-z)
Supplement: Supplementary file 1 — (DOCX 21 kb) [file 520_2019_4649_MOESM1_ESM.docx]

| **CaSUN unmet needs** | | Domain | % Endorsing | Need was fully met | Need was NOT fully met |
| --- | --- | --- | --- | --- | --- |
| 1. Up to date information. | | **IN** | 46% | 76 | 24 |
| 2. Information for my family. | | **IN** | 36% | 76 | 24 |
| 3. Information provided in a way that I can understand. | | **IN** | 52% | 84 | 16 |
| 4. The very best medical care. | | **CCC** | 68% | 91 | 9 |
| 5. Local health care services. | | **CCC** | 49% | 91 | 9 |
| 6. To feel like I am managing my health together with the medical team. | | **CCC** | 68% | 84 | 16 |
| 7. To know that all my doctors talk to each other to coordinate my care. | | **CCC** | 64% | 74 | 26 |
| 8. Concerns regarding my care to be properly addressed. | | **CCC** | 66% | 83 | 17 |
| 9. Access to complementary or alternative therapy services. | | **PC** | 29% | 60 | 40 |
| 10. Help to reduce stress in my life. | | **ES** | 35% | 48 | 52 |
| 11. Help to manage ongoing symptoms and side effects. | | **QoL** | 44% | 63 | 35 |
| 12. Help to adjust to changes in my quality of life as a result of the cancer. | | **QoL** | 33% | 57 | 43 |
| 13. Help with having a family due to fertility problems. | | **PC** | 5% | 58 | 42 |
| 14. Help to obtain and/or maintain employment. | | **PC** | 8% | 48 | 52 |
| 15. Help to find out about financial support or governmental benefits to which I am entitled. | | **PC** | 22% | 40 | 60 |
| 16. Help getting life and/or travel insurance. | | **PC** | 14% | 41 | 59 |
| 17. Help accessing legal services. | | **PC** | 11% | 48 | 52 |
| 18. More accessible hospital parking. | | **CCC** | 36% | 40 | 60 |
| 19. Help to manage my concerns about the cancer coming back. | | **ES** | 47% | 54 | 46 |
| 20. Emotional support. | | **ES** | 39% | 62 | 38 |
| 21. Help to know how to support my partner and/or family. | | **Rel** | 24% | 52 | 48 |
| 22. Help to deal with the impact that cancer has had on my relationship with my partner. | | **Rel** | 24% | 41 | 59 |
| 23. Help developing new relationships after the cancer. | | **ES** | 13% | 38 | 62 |
| 24. To talk to others who have experienced cancer. | | **ES** | 27% | 65 | 35 |
| 25. Help to handle the topic of cancer in social and/or work situations | | ES | 22% | 52 | 48 |
| 26. Help to adjust to changes to the way I feel about my body. | | **ES** | 23% | 44 | 56 |
| 27. Help to address problems with my/our sex life. | **Rel** | 22% | 24 | 76 |  |
| 28. An ongoing case manager to whom I can go to find out about services whenever they are needed. | **PC** | 33% | 30 | 70 |  |
| 29. Help to move on with my life. | **ES** | 24% | 44 | 56 |  |
| 30. Help to cope with changes to my belief that nothing bad will ever happen in my life. | **ES** | 21% | 36 | 64 |  |
| 31. Help to cope with others not acknowledging the impact that cancer has had on my life. | **ES** | 22% | 31 | 69 |  |
| 32. Help to deal with my own and/or others expectations of me as a “cancer survivor”. | **ES** | 26% | 33 | 67 |  |
| 33 Help to try to make decisions about my life in the context of uncertainty. | **ES** | 27% | 29 | 71 |  |
| 34. Help to explore my spiritual beliefs. | **ES** | 15% | 43 | 57 |  |
| 35. Help to make my life count. | **ES** | 22% | 43 | 57 |  |
